# Despair-associated memory requires a slow-onset CA1 long-term potentiation with unique underlying mechanisms

**DOI:** 10.1038/srep15000

**Published:** 2015-10-09

**Authors:** Liang Jing, Ting-Ting Duan, Meng Tian, Qiang Yuan, Ji-Wei Tan, Yong-Yong Zhu, Ze-Yang Ding, Jun Cao, Yue-Xiong Yang, Xia Zhang, Rong-Rong Mao, Gal Richter-levin, Qi-Xin Zhou, Lin Xu

**Affiliations:** 1 Key Laboratory of Animal Models and Human Disease Mechanisms, and KIZ/CUHK Joint Laboratory of Bioresources and Molecular Research in Common Disease, and Laboratory of Learning and Memory, Kunming Institute of Zoology, the Chinese Academy of Sciences, Kunming 650223, China; 2 University of the Chinese Academy of Sciences, Beijing 100049, China; 3 School of Life Sciences, University of Science and Technology of China, Hefei 230027, China; 4 School of Life Sciences, Anhui University, Hefei 230601, China; 5 Institute of Mental Health Research and Departments of Psychiatry and Cellular & Molecular Medicine, University of Ottawa, 1145 Carling Ave, Ottawa, Ontario, K1Z 7K4, Canada; 6 Sagol Department of Neurobiology and Department of Psychology, University of Haifa, Haifa, Israel; 7 The Institute for the Study of Affective Neuroscience, University of Haifa, Haifa, Israel; 8 CAS Center for Excellence in Brain Science, 320 Yue Yang Road, Shanghai, 200031, China; 9 Mental Health Institute, the Second Xiangya Hospital of Central South University, Changsha 410011, China

## Abstract

The emotion of despair that occurs with uncontrollable stressful event is probably retained by memory, termed despair-associated memory, although little is known about the underlying mechanisms. Here, we report that forced swimming (FS) with no hope to escape, but not hopefully escapable swimming (ES), enhances hippocampal α-Amino-3-hydroxy-5-methyl-4-isoxazolepropionic acid receptor (AMPAR)-dependent GluA1 Ser831 phosphorylation (S831-P), induces a slow-onset CA1 long-term potentiation (LTP) in freely moving rats and leads to increased test immobility 24-h later. Before FS application of the antagonists to block S831-P or N-methyl-D-aspartic acid receptor (NMDAR) or glucocorticoid receptor (GR) disrupts LTP and reduces test immobility, to levels similar to those of the ES group. Because these mechanisms are specifically linked with the hopeless of escape from FS, we suggest that despair-associated memory occurs with an endogenous CA1 LTP that is intriguingly mediated by a unique combination of rapid S831-P with NMDAR and GR activation to shape subsequent behavioral despair.

Stress often causes escape behaviors, neuroendocrine responses and the feeling of fear^1^. Stress may eventually lead to the feeling of despair when escape from the stressful situation is learned to be hopeless. Much like fear memory, despair congruent with the stressful situation ought to attain a privilege status in memory^2^,^3^,^4^, termed ‘despair memory’, as these emotional memories are important to shape survival strategy and behavior^5^. Extensive studies have unraveled how fear memory is processed^6^, while little is known about how ‘despair memory’ is formed. The hippocampus is critical for certain types of memory^7^,^8^,^9^, including those that contain emotionally charged information^10^. The hippocampus is particularly sensitive to stress^11^. Stress and its effects on the hippocampus have been implicated in the etiology of major depressive disorder (MDD)^12^,^13^,^14^, characterized by symptoms including despair or hopelessness. Furthermore, the hippocampus is believed to be an important action site of many antidepressants^14^,^15^. It is thus reasonable to assume that the hippocampus is involved in despair-associated memory.

Low-dose ketamine produces rapid and sustained antidepressant effect in clinic^16^,^17^. This discovery suggests the possible roles of the NMDAR-dependent synaptic plasticity and memory in the effects of the drug^14^ and pinpoints a reverse translational direction in animal studies to find out the underlying mechanisms for MDD^18^. Memory is generally believed to depend on synaptic plasticity such as LTP and long-term depression (LTD), for which NMDAR and its downstream signaling cascades including Ca2+/calmodulin-dependent protein kinase II (CaMKII) are critical^8^,^9^,^19^,^20^, a process that can be blocked by the NMDAR antagonists such as ketamine^21^. In line with that, inhibitory avoidance learning induces NMDAR-dependent LTP in hippocampal CA1 region^22^, for which the AMPAR subunit GluA1 S831-P by CaMKII activity is a prerequisite^22^,^23^,^24^. Moreover, inescapable but not escapable stress impairs CA1 LTP^25^ but facilitates CA1 LTD^26^,^27^, when hippocampal GR is activated by stress levels of glucocorticoids^28^,^29^. Furthermore, many antidepressants appear to reduce but rescue stress-impaired CA1 LTP^21^. Therefore, these findings may converge on a possibility that certain types of inescapable or uncontrollable stress may have induced an endogenous LTP in the hippocampus^4^,^30^ to process despair-associated memory, representing a particular form of the stress-induced metaplasticity^10^,^31^,^32^ to govern subsequent behavioral despair.

To address this question, we established a hopefully escapable swimming (ES) paradigm based on the forced swimming (FS) model, and defined the increase of immobility in the FS group, which was absent in the ES group, as the ‘despair-associated memory’. The formation of despair-associated memory was associated with a slow-onset endogenous LTP in hippocampal CA1 region with particular underlying mechanisms, suggesting that treatment of MDD may engage disruption of despair-associated memory.

## Results

### Uncontrollable but not controllable stress induced increase of test immobility in the forced swimming test in rats

**Figure 1:**
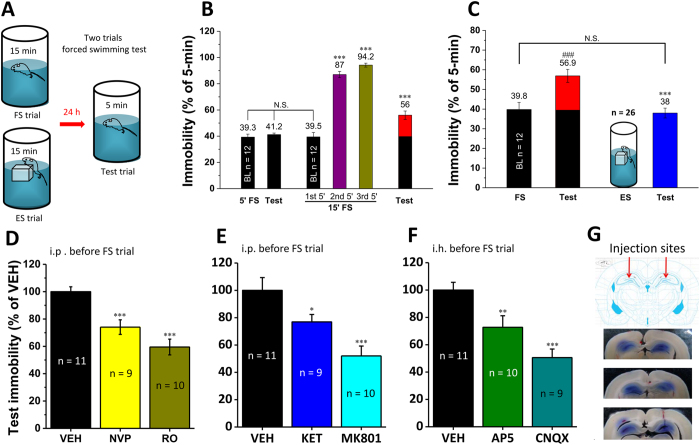
Forced swimming (FS) but not hopefully escapable swimming (ES) increased test immobility dependent on the hippocampal memory mechanisms. (**A**) Schematic graph of FS/ES and test trial. (**B**) Immobility was similar in FS and test trial for 5 min, suggesting a baseline level (BL) of immobility; immobility was greatly increased during FS trial for 15-min and the increase was largely remained during test trial, suggesting a learned increase of test immobility. *vs. BL. (**C**) A ES trial for 15-min had no effect on test immobility. #*vs.* BL; *FS vs. ES. (**D**,**E**) Intraperitoneal injection (i.p.) of the NMDAR antagonists, NVP-AAM007 (NVP), RO25-6981 (RO), ketamine (KET), MK-801 (MK801) 30 min before FS reduced test immobility. (**F**) Bilateral intrahippocampal injection (i.h.) of the NMDAR antagonist AP-5 (AP5) and AMPAR antagonist CNQX 30 min before FS reduced test immobility. (**G**) Verification of the injection sites in the hippocampus. N.S., no significant difference, *p < 0.05, **p < 0.01 and *** p < 0.001, compared with vehicle control (VEH).

In this study, we used the forced swimming (FS) test in rats, because test immobility was suggested to score a learned behavioral despair^33^, although this viewpoint has been debated^34^,^35^,^36^. Notably, we established a hopefully escapable swimming (ES) paradigm by placing a floating platform into the water (Fig. 1A), such that the rats could attempt to climb onto that unstable platform even though they were difficult to stay on it. This way, ES rats were exposed to stress similar to FS group, but with hopefully potential escape.

**Figure 2:**
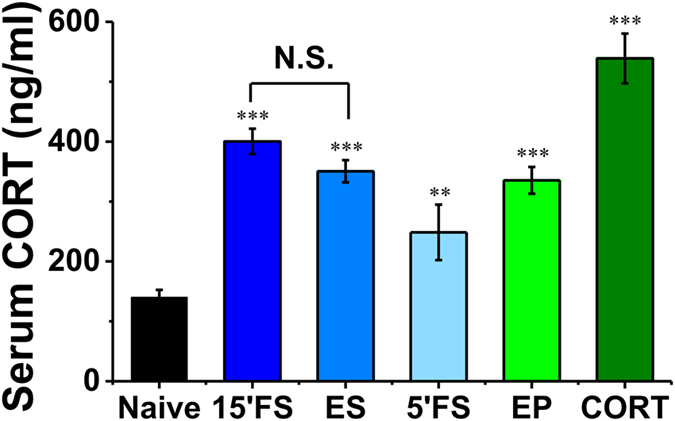
Serum corticosterone level was increased in a similar level in FS and ES groups. 30 min after the beginning of FS or ES, the serum corticosterone levels were significant higher than those of naïve group, while there was no significant difference between FS and ES group. 5 min FS trial also increased serum corticosterone level but in a lower extent. Rats exposed to elevated platform stress (EP), or treated with corticosterone (CORT, i.p., 5 mg/kg) also shown increased corticosterone levels. **p < 0.01, ***p < 0.001, compared with naïve. N.S. no significant difference.

We first measured the effects of only 5-min FS on immobility during and 24 h following the exposure. Immobility during the test on day 2 was similar to that during the 5-min FS on day 1, indicating a baseline level of immobility (BL) (Fig. 1B, n = 12, 5′FS = 39.3 ± 2.3% vs. test = 41.2 ± 1.2%, *p* = 0.45). However, immobility was significantly increased from the 1^st^ 5-min (also BL) to the end of a conventional 15-min FS trial, which was associated also with a significant increase of immobility during the 5-min test trial 24 h later (Fig. 1B, n = 12, 15′ FS: 1^st^ 5′ (BL) = 39.5 ± 3.3%, 2^nd^ 5′ = 87.0 ± 2.4%, 3^rd^ 5′ = 94.2 ± 1.5%; test = 56.0 ± 3.2%; all vs. BL, *p* < 0.001, Supplementary Figure 1 shows the original data). Importantly, such an increase of test immobility was observed in FS group (Fig. 1C, FS, n = 12, BL = 39.8 ± 3.6% vs. test = 56.9 ± 3.3%, *p* < 0.001), but it was completely absent in ES group (Fig. 1C, ES, n = 26, test = 38.0 ± 2.5%; ES vs. FS, *p* < 0.001. ES group showed the combination of two batches of data: the first one was tested parallel with Fig. 1B, n = 14, and the second batch was done independently in Fig. 1C, n = 12.), in which the immobility was similar to BL of the FS group (ES vs. BL, *p* = 0.903). Furthermore, serum corticosterone level was measured in these animals 30 min after the beginning of the FS and ES trial, and both the groups showed a similar increase of corticosterone level (Fig. 2, in ng/ml. n = 8, naïve = 140.2 ± 12.2; n = 8, 15′FS = 400.3 ± 21.1, vs. naïve, *p* < 0.001; n = 8, ES = 350.5 ± 18.5, vs. naïve, *p* < 0.001; FS vs. ES, *p* = 0.141; n = 5, 5′FS = 248.5 ± 46.3, vs. naïve, *p* = 0.007). Therefore, we demonstrated for the first time that uncontrollable (FS) but not controllable stress (ES) induced increase of test immobility 24-h after training, although both treatments increased the major stress hormone to a similar level.

### Increase of test immobility was a type of despair-associated memory dependent on hippocampal glutamate transmission

Reduced test immobility by giving substances before test trial is widely used to evaluate their antidepressant efficacy^33^. In accordance with our proposed model, if increased test immobility indicates a type of plasticity-dependent memory, blockade of NMDAR during FS could prevent increased test immobility. We tested the effects of the NMDAR antagonists, NVP-AAM077 (NVP, 1.2 mg/kg) or Ro 25-6981 (RO, 6 mg/kg) or ketamine (KET, 15 mg/kg, a subanesthetic dose in rats) or MK-801 (MK801, 0.1 mg/kg) given intraperitoneally (i.p.) 30 min before FS. In relative to vehicle control (VEH), the NMDAR antagonist drugs significantly reduced test immobility (Fig. 1D, n = 11, VEH = 100 ± 3.6%; n = 9, NVP = 74.1 ± 5.3%, vs. VEH, *p* = 0.001; n = 10, RO = 59.5V5.7%, vs. VEH, *p* < 0.001; Fig. 1E, n = 12, VEH = 100 ± 9.4%; n = 12, KET = 76.9 ± 5.4%, vs. VEH, *p* < 0.001; n = 12, MK801 = 52.0 ± 7.2%, vs. VEH, *p* < 0.001) to levels similar to ES group in Fig. 1C (n = 26, 65.2 ± 4.3% of VEH). To verify specifically the role of CA1, we further tested the effects of glutamate receptors blockade directly within CA1. Drugs known to block NMDAR or AMPAR were infused bilaterally into hippocampal CA1 regions (intrahippocampal injection, i.h., 1 μl/side) 30 min prior to FS. The AMPAR antagonist 6-cyano-7-nitroquinoxaline-2,3-dione (CNQX, 3 mM) or NMDAR antagonist AP-5 (AP5, 30 mM) significantly attenuated test immobility (Fig. 1F, n = 11, VEH = 100 ± 5.7%; n = 10, AP5 = 72.7 ± 8.5%, vs. VEH, *p* = 0.008; n = 9, CNQX *=* 50.6 ± 6.3%, vs. VEH, *p* < 0.001; Fig. 1G, verification of the i.h. injection sites). Thus, increase of test immobility induced by FS could be a type of memory associated with despair, and it was dependent on the hippocampal AMPARs and NMDARs.

### Despair-associated memory was linked with phosphorylation of the synaptic AMPAR GluA1 Ser831

**Figure 3:**
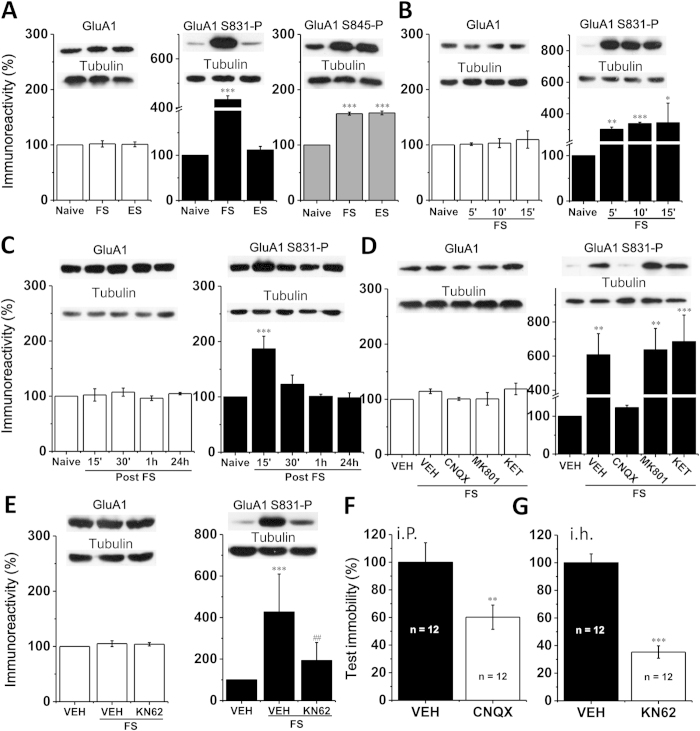
FS but not ES increased AMPAR/CaMKII activity-dependent GluA1 Ser831 phosphorylation (S831-P) in the hippocampus. (**A**) Neither FS nor ES affected synaptic GluA1 expression. However, FS greatly increased synaptic S831-P and GluA1 Ser845 phosphorylation (S845-P), but ES increased synaptic S845-P only, immediately after FS/ES. (**B**) S831-P was rapidly increased within about 5-min during FS. (**C**) Increase of S831-P was maintained for about 30-min and decayed to Naïve level at 1 and 24 h after FS. (**D**) Intraperitoneal injection (i.p.) of the AMPAR antagonist CNQX but not NMDAR antagonists MK801 or KET 30 min before FS prevented increase of S831-P, and (**F**) CNQX also prevented increase of test immobility. (**E**,**G**) CaMKII inhibitor KN-62 (KN62) prevented not only increase of S831-P but also increase of test immobility. In B-E, synaptic GluA1 expression remained unchanged. *p < 0.05, **p < 0.01, ***p < 0.001, compared with Naïve or vehicle control (VEH).

LTP and some forms of memory are believed to depend on the GluA1 S831-P (CaMKII/protein kinase C site)^24^ but not Ser845 phosphorylation (S845-P, protein kinase A site)^22^,^23^. Thus, we examined whether FS/ES influenced hippocampal synaptic S831-P and S845-P, whether this was dependent on AMPAR and NMDAR, and whether it was critical for the observed increase of test immobility. Synaptoneurosomes were prepared from the rat hippocampus after FS/ES. Synaptic GluA1 expression was not modified by either FS or ES (Fig. 3A, GluA1, n = 5; FS = 101.8 ± 5.6%, vs. Naïve, *p* = 0.78; ES = 101.1 ± 4.4%, vs. Naïve, *p* = 0.8). Both synaptic S831-P and S845-P were greatly increased when examined immediately after FS in relative to Naïve (Fig. 3A, FS group, n = 5; S831-P = 433.4 ± 15.5%, vs. Naïve, *p* < 0.001; S845-P = 156.6 ± 2.7%, vs. Naïve, *p* < 0.001). In contrast, in the ES group only was synaptic S845-P largely increased (Fig. 3A, ES group, n = 5; S831*-*P = 111.6 ± 8.1%, vs. Naïve, *p* = 0.56; S845-P = 157.9 ± 3.4%, vs. Naïve, *p* < 0.001). Thus, S831-P was specifically increased by FS but not ES. Furthermore, we found that S831-P was significantly increased over 15-min FS trial (Fig. 3B, S831-P, n = 3; 5′ = 301.8 ± 13.8%, vs. Naïve, *p* = 0.005; 10′ = 337.7 ± 8.6%, vs. Naïve, *p* = 0.001; 15′ = 343.2 ± 134.4%, vs. Naïve, *p* = 0.045). This increase of S831-P lasted for about 30-min and restored to Naïve level at 1 and 24 h post FS (Fig. 3C, post-FS, S831-P, n = 4; 15′ = 188.6 ± 19.8%, vs. Naïve, *p* < 0.001; n = 5, 30′ = 122.7 ± 16.8%, 1 h = 101.0 ± 3.9%, 24 h = 98.3 ± 9.1%, vs. Naïve, *p* = 0.21, 0.96 and 0.93, respectively). Synaptic S845-P was also rapidly and transiently increased and GluA1 expression remained unchanged during this time period (Fig. 3B, all groups, n = 3; Fig. 3C, 15′, n = 4; other groups, n = 5; all groups vs. Naïve, *p* > 0.05. For S845-P, see Supplementary Figure 3). Meanwhile, during the ES trial the synaptic S831-P level remained unchanged (Supplementary Figure 2). These findings suggested that FS induced a short-lifetime S831-P specifically.

### AMPAR and CaMKII activity-dependent S831-P was required for despair-associated memory

We then examined whether the synaptic S831-P increased by FS specifically was dependent on AMPAR or NMDAR. Rats were treated (i.p.) with CNQX (2 mg/kg), MK801 (0.1 mg/kg) or KET (15 mg/kg) 30 min before FS, and synaptoneurosomes were prepared immediately after FS. We found that the increase of S831-P was completely blocked by CNQX, but surprisingly unaffected by MK801 or KET (Fig. 3D, S831-P, all groups n = 4; VEH + FS = 607.7 ± 124.1%, vs. VEH, *p* = 0.004; CNQX + FS = 122.8 ± 6.3%, vs. VEH, *p* = 0.184; MK801 + FS = 637.1 ± 125.0%, vs. VEH, *p* = 0.003; KET + FS = 685.1 ± 155.2%, vs. VEH, *p* = 0.001). This CNQX treatment (i.p., 2 mg/kg) also inhibited test immobility (Fig. 3F, n = 12, VEH = 100 ± 14.1%; n = 12, CNQX = 60.2 ± 8.8%, vs. VEH, *p* = 0.009). To further confirm whether this increase of synaptic S831-P was critical for the FS-induced increased test immobility, we applied CaMKII inhibitor KN-62 (KN62, 25 μg/5 μl, i.c.v. for S831-P test; 1 μg/1 μ/side, i.h. for behavior test) 30 min before FS. KN62 largely reduced S831-P (Fig. 3E, S831-P; n = 6, VEH; n = 6, FS + VEH = 427.0 ± 182.7%, vs. VEH, *p* < 0.001; n = 8, FS + KN62 = 192.9 ± 87.0%, vs. VEH, *p* = 0.149, vs. FS + VEH, *p* = 0.002) and inhibited test immobility (Fig. 3G, n = 12, VEH = 100 ± 6.4%; n = 12, KN62 = 35.3 ± 4.5%, vs. VEH, *p* < 0.001, and see also Supplementary Figure 4). In contrast, application of the protein kinase A inhibitor H89 affected neither FS-induced S831-P nor test immobility (Supplementary Figure 5). These results indicated that uncontrollavble but not controllable stress enhanced AMPAR and CaMKII activity-dependent S831-P to be critical for despair-associated memory.

### Despair-associated memory depended on the NMDAR-mediated endogenous LTP in hippocampal CA1

**Figure 4:**
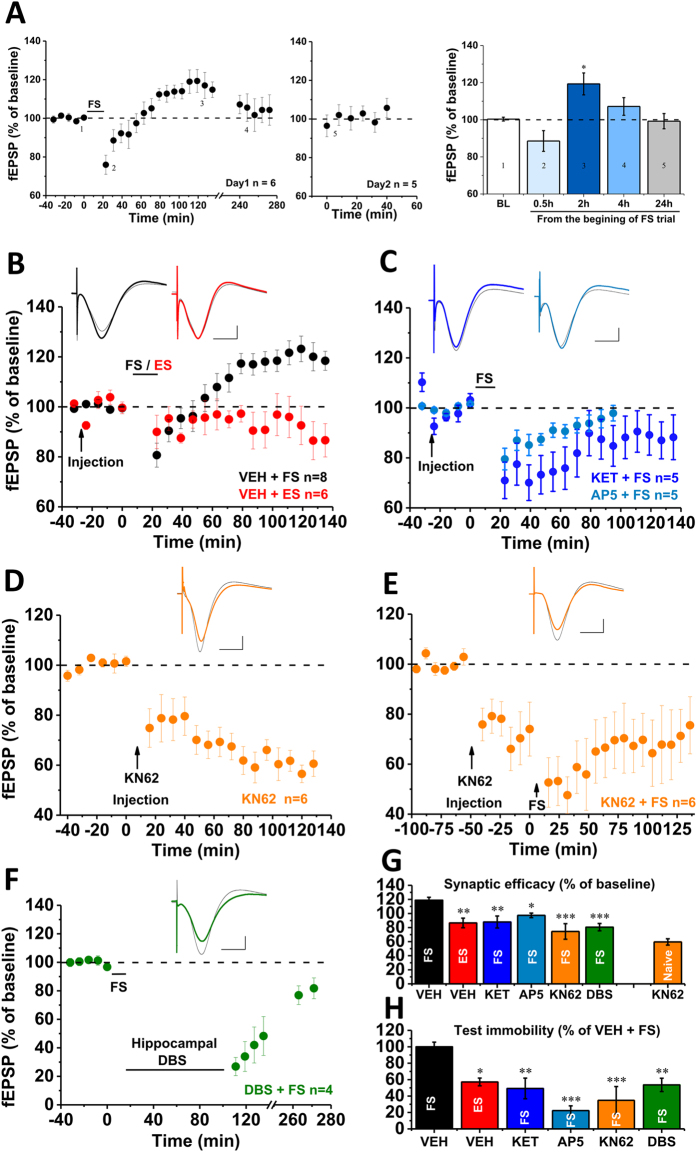
FS but not ES induced an endogenous CA1 LTP in freely moving rats. (**A**) FS induced a slow-onset CA1 LTP, started by synaptic depression, and followed by reversal of the depression to LTP by 1 h, and maintained for about 4 h. (**B**) FS induced a reliable CA1 LTP, but ES had no effect on the fEPSP. (**C**) Intracerebroventricular injection (i.c.v.) of AP-5 (AP5) or intraperitoneal injection (i.p.) of ketamine (KET) 30 min before FS prevented the CA1 LTP. (**D**) A selective CaMKII inhibitor KN-62 (KN62, i.c.v.) produced a synaptic depression lasting for several hours, (**E**) during which the CA1 LTP was prevented. (**F**) Deep brain stimulation (DBS) applied via the stimulating electrode prevented the CA1 LTP. (**G**,**H**) Summary of electrophysiological and behavioral data, suggesting a possible causal relation between the CA1 LTP and reduced test immobility. *p < 0.05, **p < 0.01, ***p < 0.001, compared with FS group or vehicle control (VEH). Scale bars for the fEPSP traces: 10 ms and 1 mV.

We directly addressed the possibility that FS could induce an endogenous CA1 LTP to underlie the FS-induced despair-associated memory^26^,^27^. We recorded the field excitatory postsynaptic potentials (fEPSP) from the Schaffer/commissural-CA1 pathway in freely moving rats^26^ before and after FS/ES, and measured test immobility in these animals. FS induced a slow-onset synaptic potentiation in CA1, with an initial and transient synaptic depression and fully reversed to LTP within about 40–60 min and lasted for about 4 h (thus an endogenous slow-onset CA1 LTP) (Fig. 4A, Day 1 n = 6, BL = 100.3 ± 1.0%; 0.5 h = 88.5 ± 5.6%; *2 h = 119.3 ± 5.9%; 4 h = 107.6 ± 4.8%; Day 2 n = 5, 24 h = 99.2 ± 4.1%; vs. BL, *p* = 0.103, *0.031, 0.175 and 0.645 respectively). Remarkably, while FS induced a reliable LTP (Fig. 4B, n = 8, VEH + FS = 118.8 ± 4.1%, vs. baseline, *p* = 0.003), ES had no significant effect on synaptic efficacy (Fig. 4B, n = 5, VEH + ES = 86.6 ± 6.8%, vs. baseline, *p* = 0.234; FS vs. ES, *p* = 0.001), indicating also that other potential influences of FS/ES such as body temperature changes may be unrelated to the synaptic potentiation. Furthermore, this slow-onset synaptic potentiation was found to be a form of NMDAR-dependent CA1 LTP, because the NMDAR antagonists KET (i.p. 15 mg/kg) or AP5 (i.c.v., 10 mM) blocked it without affecting the transient synaptic depression (Fig. 4C, n = 5, AP5 = 97.3 ± 2.2%; n = 5, KET = 87.9 ± 8.5%; AP5 or KET vs. baseline, *p* = 0.315 or 0.102). Thus, controllable stress had no significant effect on synaptic efficacy and test immobility but uncontrollable stress induced CA1 LTP and increased test immobility (Fig. 4H), both of which were blocked by NMDAR antagonists, landing further support to the new concept that the CA1 LTP may underlie despair-associated memory that leads to despair-like behavior.

### Blockade of the endogenous CA1 LTP blocked despair-associated memory

To further examine whether S831-P is a prerequisite to induce the CA1 LTP and increase of test immobility in the same rats, we administered CaMKII inhibitor KN62 (25 μg/5 μl, i.c.v.) after 40-min baseline recordings. KN62 significantly reduced basal fEPSP, resulting in a synaptic depression which lasted for several hours (Fig. 4D, n = 6, KN62 = 59.6 ± 4.5%, vs. baseline, *p* = 0.002), during which KN62 prevented FS to induce the CA1 LTP (Fig. 4E, n = 6, pre-FS = 73.1 ± 10.5% vs. KN62 = 74.5 ± 11.1%, *p* = 0.855) and reduced test immobility (Fig. 4H). As a yet additional test to the association between the CA1 LTP and despair-associated memory we examined the effect of deep brain stimulation (DBS). DBS is found to disrupt memory and is used in recent years to treat treatment-resistant MDD in patients^37^. DBS applied to the hippocampal CA1 area via the stimulating electrode blocked the CA1 LTP (Fig. 4F, n = 4, DBS = 80.6 ± 5.2%, vs. baseline, *p* = 0.091) and reduced test immobility (Fig. 4H); sham stimulation had no significant effect on test immobility (n = 6, 115.9 ± 20.8% of VEH + FS). We summarized the electrophysiological and behavioral data, because the accumulating evidence strongly supports a possible causal relation between the CA1 LTP (Fig. 4G, compared with VEH + FS: VEH + ES, *p* = 0.001; KET + FS, *p* = 0.001; AP5 + FS, *p* = 0.02; KN62 + FS, *p* < 0.001; DBS + FS, *p* = 0.001) and test immobility (Fig. 4H, n = 8, VEH + FS = 100 ± 5.6%; n = 6, VEH + ES = 57.2 ± 4.7%, *p* = 0.002; n = 5, KET + FS = 49.3 ± 12.6%, *p* = 0.001; n = 5, AP5 + FS = 22.3 ± 5.8%, *p* < 0.001; n = 6, KN62 + FS = 34.8 ± 16.8%, *p* < 0.001; n = 7, DBS + FS = 53.6 ± 8.1%, *p* = 0.005; all compared with VEH + FS group).

The theory of metaplasticity provides the prediction of the consequences of the FS-induced CA1 LTP, such as occlusion of the subsequent LTP induction by using high-frequency stimulation (HFS)^26^,^27^. Just as the prediction, we found that HFS failed to induce CA1 LTP in FS rats, that showed increase of test immobility, but CA1 LTP could be induced in ES rats that did not exhibit increase of test immobility (Supplementary Figure 6). These findings are compatible with the notion that uncontrollable but not controllable stress may induce an endogenous form of CA1 LTP which is critical for the formation of despair-associated memory, mediating not only the increase of test immobility but also the subsequent influences on synaptic plasticity.

### Activation of GR by stress levels of corticosterone was required for both the endogenous CA1 LTP and despair-associated memory

**Figure 5:**
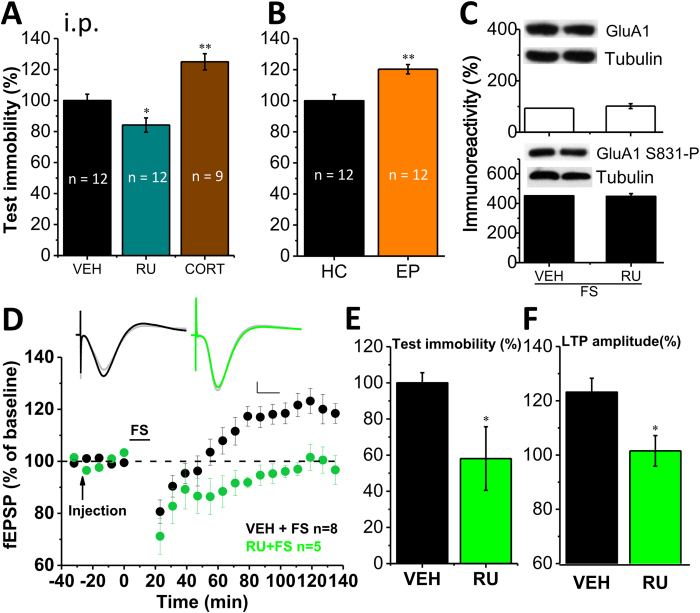
CA1 LTP and increase of test immobility were also dependent on corticosterone (CORT)/glucocorticoid receptors (GR). (**A**) Intraperitoneal injection (i.p.) of the GR antagonist RU38486 (RU) or CORT 30 min before FS reduced or enhanced test immobility, respectively. (**B**) Elevated-platform (EP) stress but not homecage (HC) exposure for 30-min before FS led to a greater increase of test immobility. (**C**) Neither synaptic GluA1 expression nor GluA1 S831-P was affected by RU treatment 30-min before FS. (**D**,**F**) RU treatment 30-min before FS disrupted the CA1 LTP and (**E**) reduced test immobility. *p < 0.05, **p < 0.01, ***p < 0.001, compared with vehicle control (VEH). Scale bars for the fEPSP traces: 10 ms and 1 mV.

The FS-induced CA1 LTP developed about 40–60 min after FS, a time period that is sufficient for hippocampal GR activation by the stress-triggered release of corticosterone^38^. We thus performed experiments to test whether the slow-onset CA1 LTP and despair-associated memory were also dependent on GR activation. Animals were treated (i.p.) with the GR antagonist RU38486 (RU, 15 mg/kg) or exogenous corticosterone (CORT, 5 mg/kg) 30 min before FS. Test immobility was significantly reduced by RU, but it was further increased by corticosterone (Fig. 5A, n = 9, RU = 84.2 ± 4.6%, vs. VEH, *p* = 0.049; n = 9, CORT = 125.0 ± 5.2%, vs. VEH, *p* < 0.001; Fig. 2, CORT, n = 5, serum CORT level 30 min after exogenous CORT treatment, 538.9 ± 41.5 ng/ml, vs. naïve, *p* < 0.001), suggesting that GR might play important roles in despair-associated memory. The application of the mineralocorticoid receptor (MR) antagonist spironolactone (SPI, i.p. 100 mg/kg) or β-adrenergic receptor blocker propranolol (PROP, i.p. 20 mg/kg) 30 min before FS had no effect on test immobility (SPI, n = 9, 108.2 ± 10.1% of VEH, SPI vs. VEH, *p* = 0.56; PROP, n = 10, 94.6 ± 11.0% of VEH, PROP vs. VEH, *p* = 0.738). We further examined whether raising endogenous corticosterone levels towards FS by additional stress could increase test immobility. Rats were exposed to an elevated platform (EP) for 30 min, known as a mild stress to induce stress levels of corticosterone^26^ (Fig. 2, n = 8, EP = 335.3 ± 22.3 ng/ml, vs. naïve, *p* < 0.001), immediately before FS. EP exposure resulted in a greater increase of test immobility relative to homecage (HC) exposure (Fig. 5B, n = 10, EP = 120.3 ± 3.0%, HC vs. EP, *p* = 0.002). However, neither synaptic GluA1 expression nor S831-P were affected by RU treatment 30 min before FS (Fig. 5C, VEH + FS group is set at the level of that in Fig. 3A. RU + FS, n = 4, GluA1 = 101.5 ± 9.5%, vs. VEH + FS, *p* = 0.86; S831-P = 448.0 ± 18.4%, vs. VEH + FS, *p* = 0.07), suggesting that the role of GR, similar to that of NMDAR, is either downstream to or has a parallel path of action to that of AMPAR and CaMKII-dependent S831-P, though the both are required for the formation of despair-associated memory. Furthermore, we directly examined whether GR activation was critical for the CA1 LTP and increase of test immobility in the same animals. RU treatment 30 min before FS blocked the CA1 LTP without affecting the short synaptic depression (Fig. 5D,F, VEH + FS, data from Fig. 4B; n = 5, RU + FS = 101.5 ± 5.6%, vs. baseline, *p* = 0.322; VEH vs. RU, *p* = 0.012) and reduced test immobility (Fig. 5E, n = 8, VEH + FS; n = 5, RU + FS = 58.1 ± 17.6%, VEH vs. RU, *p* = 0.02), to levels similar to those of the ES group. In marked contrast, if applied immediately after FS, neither corticosterone nor RU treatment affected increase of test immobility as compared to VEH (VEH, n = 12, 100 ± 6.2%; CORT, n = 11, 96.9 ± 9.4%; RU, n = 10, 86.8 ± 7.2%; CORT vs. VEH, *p* = 0.775; RU vs. VEH, *p* = 0.236), indicating a strict timing window between FS trial and GR activation for the formation of despair-associated memory.

## Discussion

The FS/ES protocol we employed here was effective for studying the underlying mechanisms of despair-associated memory that might shape cognition and behavior in the future. We found that animals in FS/ES groups shared similarities such as behavioral signs of stress (such as defecation), corticosterone levels (Fig. 2), an increase of S845-P and a transient synaptic depression. Despite that, were only in the FS group an increase in S831-P and a slow-onset CA1 LTP found. Most importantly, was only in the FS group a subsequent elevated immobility observed during the test, in agreement with the concept of the controllable and uncontrollable stress^26^,^39^,^40^. These findings are consistent with a recent report indicating that several types of inescapable stress including FS trial for 20-min increased NMDAR- and AMPAR-mediated excitatory postsynaptic currents in rat prefrontal cortex when GR was activated about 1 h after the stress exposure^41^. Thus under the uncontrollable situation, the AMPAR and CaMKII activity-induced rapid GluA1 phosphorylation, NMDAR and GR activation may work together to induce the slow-onset CA1 LTP that underlies despair-associated memory.

The different outcomes of FS and ES pinpoint the necessity of the uncontrollable stress nature in the formation of despair-associated memory. GluA1 S831-P may serve as a synaptic tagging for the subsequent molecular events in the selected synapses, because the increase of S831-P in FS group is independent from NMDAR and GR activation (Figs 3D and 5C). This disassociation between the synaptic tagging and the CA1 LTP is highly consistent with the synaptic tagging and capture hypothesis for the cellular consolidation of memory^42^. In particular, S831-P, NMDAR and GR were required for both the CA1 LTP and increased test immobility, suggesting that the molecular events could be bridged by the synaptic tagging mechanisms. It has been reported that GluA1 S831-P is involved in encoding emotional significance, facilitating LTP induction and enhancing emotional memory under stress situation^22^,^43^. So despair situation that have induced the synaptic tagging and the following LTP may be a major contributor to subsequent synaptic dysfunctions and behavioral abnormalities^25^,^26^,^27^,^44^,^45^.

**Figure 6:**
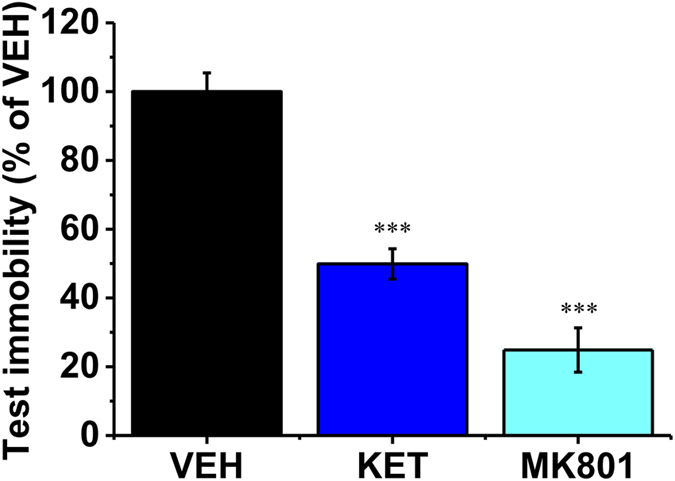
Ketamine or MK801 administrated before test trial blocked the retrieval of despair-associated memory. Vehicle (VEH), ketamine (KET) or MK801 was treated 30 min before test trial. In groups treated with ketamine (KET, i.p. 15 mg/kg) or MK801 (i.p. 0.1 mg/kg), the immobility was greatly reduced. ***p < 0.001.

**Figure 7:**
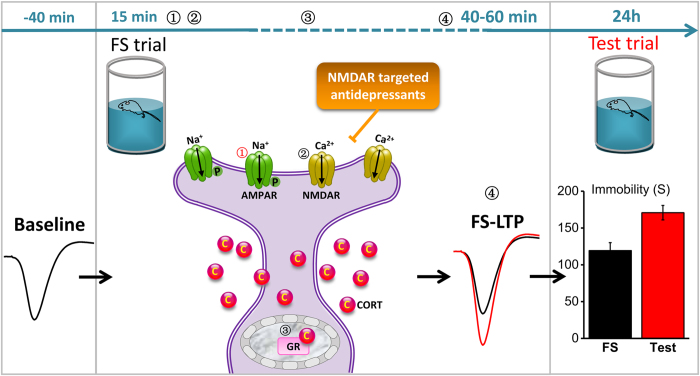
Schematic model for the process of despair-associated memory. FS induced a rapid increase of GluA1 S831P①, followed by the conjunctive activation of NMDAR② and GR③. These molecular events led to an endogenous CA1 LTP④, and contributed to despair-associated memory (increase of test immobility). According to this model, antidepressants that target on AMPAR, NMDAR (such as ketamine) and GR may exert antidepressant effect by preventing the formation and retrieval of despair-associated memory.

Many of the hypotheses for MDD have proposed that chronic and mild stressful experience must have certain contributions to the development of the disorder^13^,^14^,^35^,^46^,^47^,^48^. The powerful memory mechanisms for emotionally charged information would be highly conserved in evolution because they are critical in shaping survival cognition and behavior^5^. Here, we find that the uncontrollable type of acute stress induces a slow-onset CA1 LTP that underlies despair-associated memory. Given the unique mechanisms of the despair memory we demonstrated here, it is possible that the effects of chronic mild stresses on emotion could be accumulated by the memory mechanisms and thus likely led to psychopathological changes. In addition, the retrieval of despair-associated memory was prevented by NMDAR antagonists when treated 30 min before test trial (Fig. 6, n = 12, VEH = 100 ± 5.45%; n = 12, KET = 49.9 ± 4.4%, vs. VEH, p < 0.001; n = 12, MK801 = 24.9 ± 6.4%, vs. VEH, p < 0.001). Such a possible relation of despair memory mechanisms with MDD is also implicated by the antidepressant effects of ketamine, because a single treatment of ketamine reduced test immobility and the effect was maintained for about 7 d^49^, similar to the clinical features of ketamine in treating MDD patients^16^. Furthermore, the DBS treatment, which is known to disrupt memory and produces antidepressant efficacy in clinic, disrupted the uncontrollable stress-induced CA1 LTP and despair-associated memory. Thus, as the schematic diagram shown in Fig. 7, our evidencefor the hippocampal mechanisms of the ‘despair memory’ formation and retrieval should thus provide a new insight into not only the development of the stress-induced MDD but also the prevention and treatment of such psychopathologies.

## Materials and Methods

### Animals

Male Sprague Dawley rats (Animal Housing Center, Kunming Medical University, Kunming, China) weighing 250–300 g were used. Animals were group housed, but single housed after surgery, in ventilated cages with free access to water and food in a 12-h light/dark cycle and temperature-regulated environment in the Animal Facilities of Kunming Institute of Zoology (the Chinese Academy of Sciences, Kunming 650223, China). All experiments were carried out in accordance with the approved guidelines of ethics committee of Kunming Institute of Zoology, and all experimental protocol were approved by ethics committee of Kunming Institute of Zoology, Chinese Academy of Sciences.

### Drugs

All reagents are purchased from Sigma-Aldrich (St. Louis, Missouri, USA), except for NVP-AAM0077 (NVP, from Prof. Xia Zhang, Ottawa, Canada); KN-62 (KN62, from ABCAM, Cambridge, UK); spironolactone and RU38486 (SPI and RU, from Yuancheng Technology Development Co. Ltd., Wuhan, China); ketamine (KET, from Fujian Gutian Pharmaceutical Co. Ltd., Gutian, Fujian, China). Corticosterone (CORT), RU, KN62 and SPI were dissolved in dimethyl sulfoxide (DMSO). The others were dissolved in sterile saline.

### The forced swimming (FS) test and hopefully escapable swimming (ES)

The experimental protocols were modified from those described previously^33^,^50^. Briefly, rats were allowed to adapt to test room for 1 h before FS or test trial. All experiments were conducted between 8:30 and 18:00. The apparatuses were glass cylinders, 50 cm in height and 26 cm in diameter, and filled with 35 cm in height of tap water at 25 ± 1 °C that was the same as the regulated-room temperature. A hopefully escapable swimming (ES) paradigm was established by placing a floating platform into the water (Fig. 1A). This floating platform was a waterproof plastic box, 12 cm length, 10 cm width, 9 cm height, and stones were fixed to the inside bottom of the box to reach a final weight around 950 g. The floating platform was placed into the glass cylinders filled with water during ES trial for 15-min, but it was removed during test trial 24 h later. Notably, this floating platform was unstable, as ES rats could attempt to climb onto it but they were actually difficult to stay on it. After swimming, rats were dried very carefully by using a towel before returned to their homecages or the recording chamber for electrophysiological studies. Immobility was scored by skillful technicians to measure the accumulative time of the rats spent in immobile (slight movements of limbs that are necessary for keeping nose above the water are also account as immobility). Since there was no behavior equivalent to immobile posture in the ES trial on day 1, the immobility could be measured only during the test trial on day 2 in the ES group when the platform was removed. Each individual behavioral data were normalized (%) to the mean value of control group and then the mean and SEM were further calculated, except for the behavioral data in Fig. 1B,C which were normalized (%) to 5-min.

### Surgery and drugs injection

Under pentobarbital sodium anesthesia (i.p., 60 mg/kg), rats were implanted with stainless steel guide cannulas (26 gauge, 11 mm, from RWD Life Science Co. Ltd., Shenzhen, Guangdong, China) that were affixed to the skull with dental cement by using techniques similar to those described^26^,^51^,^52^,^53^. The cannulas were located into the lateral cerebroventricle (0.1 mm caudal of the Bregma, 1.6 mm lateral from the midline and 4.0 mm depth from the brain surface) or the bilateral hippocampal CA1 areas (3.8 mm caudal of the Bregma, 2.8 mm lateral from the midline and 2.2 mm depth from the brain surface). Intracerebroventricular (i.c.v.) or i.h. (i.h.) injections were made over a 6-min period by using a syringe pump (LSP02-1B Dual Channels Syringe Pump, from Longer Precision Pump Co. Ltd, Baoding, Hebei, China), connected to injectors (32 gauges, from RWD Life Science Co. Ltd., Shenzhen, Guangdong, China) by polyethylene tubing. Except mentioned elsewhere, all drugs (including i.p., i.h., i.c.v.) were delivered 30 min before FS trial.

### Electrophysiological studies

Experimental protocols for the recordings of the field excitatory postsynaptic potentials (fEPSP) in the Schaffer/commissural-CA1 pathways *in vivo* are similar as those described previously^26^,^53^,^54^,^55^. Briefly, Implantation of the electrodes was performed in the rats under pentobarbital sodium (60 mg/kg, i.p.) anesthesia and body temperature was maintained at 37 ± 0.5 °C by a heating blanket. Three stainless-steel screws (one serving as a reference electrode, the second acting as a ground electrode and all of three serving as anchors) were inserted into the skull through drilled holes without piercing the dura. Electrodes were made by gluing together a pair of twisted Teflon-coated 90% platinum/10% iridium wires (50-μm bare diameter, 100-μm coated diameter, World Precision Instruments, Sarasota, Florida, USA) and were placed into the CA1 area at the following coordinates: 3.8 mm caudal of the Bregma, 2.8 mm lateral from the midline and 2.4 mm depth from the brain surface for the recording electrode; 4.8 mm caudal of the Bregma, 3.8 mm lateral from the midline and 2.4 mm depth for the stimulating electrode. The optimal depth of the electrodes was determined by electrophysiological criteria and was verified by post-mortem examination. For the fEPSP recordings in anaesthetized rats, after 40 min stable baseline recordings, a high-frequency stimulation (HFS, 200 Hz, 10 trains × 20 pulses/train, intertrain interval 2 s) at the baseline stimulation intensity was delivered. For the fEPSP recordings in freely moving rats and the deep brain stimulation (DBS), the entire assembly of the electrodes was sealed and fixed to the skull using dental acrylic. The rats were then placed individually into their homecages for at least 2 weeks for recovery. DBS was applied through the stimulating electrode using a clinically used protocol (100 μs pulse widths, 150 μA intensity, 130 Hz for 1.5 h) described previously^37^,^56^.

### Immunoblot assays

The synaptic proteins were made from filtered synaptoneurosomes that prepared from hippocampal tissue as those described previously^22^,^57^. The hippocampal samples were obtained in experimental or naïve rats. Dissections of the hippocampus were performed using ice-cold phosphate buffered saline (PBS) rapidly, and the samples were homogenized in ice-cold homogenization buffer (10 mM Hepes, 1.0 mM EDTA, 2.0 mM EGTA, 0.5 mM DTT, 1 mM PMSF, 10 mg/liter leupeptin, 1 mg/liter pepstatin A, 10 mM NaF, 1 mM Na_3_VO_4_, 0.2 mM β-glycerophosphate). The homogenization was performed by glass/glass tissue homogenizers, and the homogenate was passed through two 100-μm-pore nylon mesh filters (Millipore, NY1H02500), and then through a 5-μm-pore filter (Millipore, SLSV025NB). The filtered homogenates were centrifuged at 1000 g for 15 min at 4 °C. Resultant pellets (filtered synaptoneurosomes) were resuspended in 350 μL boiling 1% SDS, boiled for 5 min. After 20 μL of each sample being saved for BCA assay, the rest was mixed with 4x Laemmli Buffer in a 3:1 ratio, heated at 80 °C for 15 min, then stored at −80 °C^58^.

Immunoblot analysis was performed as previously described^59^. Synaptic protein samples (40 μg/lane) were size-separated by electrophoresis in SDS-PAGE (10% acrylamide) and transferred to PVDF membranes (Immobilon^TM^-P PVDF membrane, from Millipore Co, Billerica, Massachusetts, USA). Membranes were blocked at room temperature for 120 min with TBS-T (0.9% NaCl, 10 mM tris, 0.1% tween-20, PH7.4) containing 1% BSA on an orbital shaker. After blocking, the membrane was reacted overnight at 4 °C with the primary antibody diluted in TBS-T containing 1% BSA (Rabbit × GluA1, 1: 5000, from Millipore Co, Billerica, Massachusetts, USA; Rabbit × GluA1 S831p, 1:15000, Rabbit × GluA1 S845p, 1:15000, from Epitomics Co., Burlingame, California, USA; Mouse ×β-tubulin, 1:30,000, from CWbiotech Co. Ltd., Beijing, China). After four washes of 10 min each with TBS-T, the membranes were subsequently incubated for 2 h at room temperature with HRP-linked secondary antibody diluted in TBS-T (Goat × Rabbit/Mouse, HRP-linked, 1:20000, KangChen Bio-tech Inc., Shanghai, China). The membrane was washed for another four times before exposure to the chemiluminescent HRP substrate (Luminata^TM^ HRP substrates, from Millipore Co, Billerica, Massachusetts, USA). The light emitted by ECL reagent is subsequently captured on X-ray film (FUJIFILM Super RX, from Fuji Photo Film Co Ltd, Tokyo, Japan). Films were scanned by an Epson scanner. Then the images were transferred to grayscale and the bands intensity were analyzed by NIH ImageJ software.

### Elevated platform stress

A mild stress was evoked by elevated platform (EP) as described previously^26^,^52^. Briefly, animals were placed on an elevated platform (10 cm × 10 cm × 1.6 m) in the middle of a bright room for 30 min, and signs of stress such as freezing, defecation and urination were observed. Immediately after the EP or homecage exposure, the rats were subjected to FS.

### Serum preparation and corticosterone immunoassay

Serum preparation and corticosterone determination was performed as described previously^44^. Briefly, blood was collected by cardiac puncture under pentobarbital sodium (60 mg/kg, i.p.) anesthesia, 15 minutes after 15′FS, ES, 5′FS, 30 minutes after corticosterone injection (5 mg/kg, i.p.), or immediately after 30′ exposed to elevated platform stress. Samples were left undisturbed for at least 1 hour, then centrifuged at 1000 g twice to remove the clot. Approximately 0.5 ml of serum from each rat was collected into 1.5 ml Eppendorf tubes and stored at −80 °C. Corticosterone concentration was assessed using ENZO ADI-900-097 ELISA kits (from ENZO life science, New York, USA). The sensitivity of the corticosterone assay was 27 pg/mL.

### Statistical Analysis

Paired or unpaired Student’s *t* test or a one-way ANOVA followed by *post hoc* analysis with least significant difference (LSD) was used for comparisons. The significance level was set at *p* < 0.05. All values were reported as mean ± SEM.

## Additional Information

**How to cite this article**: Jing, L. *et al.* Despair-associated memory requires a slow-onset CA1 long-term potentiation with unique underlying mechanisms. *Sci. Rep.* **5**, 15000; doi: 10.1038/srep15000 (2015).

## Supplementary Material

Supplementary Figures
