# Supplementary material for: Despair-associated memory requires a slow-onset CA1 long-term potentiation with unique underlying mechanisms
Source: Sci Rep. 2015 Oct 9;5:15000. doi: 10.1038/srep15000 (PMC4598857; doi:10.1038/srep15000)
Supplement: Supplementary Figures [file srep15000-s1.doc]

**Supplementary Information**

Despair-associated memory requires a slow-onset CA1 long-term potentiation with unique underlying mechanisms

Liang Jing^1,2*^, Ting-Ting Duan^1,3*^, Meng Tian^1,2*^, Qiang Yuan^1,2^, Ji-Wei Tan^1,2^, Yong-Yong Zhu^1,3^, Ze-Yang Ding^1,4^, Jun Cao^1,2^,Yue-Xiong Yang^1,2^, Xia Zhang^5^, Rong-Rong Mao^1,2^, Gal Richter-levin^6,7#^, Qi-Xin Zhou^1,2#^ Lin Xu^1,2,8,9#^

^1^Key Laboratory of Animal Models and Human Disease Mechanisms, and KIZ/CUHK Joint Laboratory of Bioresources and Molecular Research in Common Disease, and Laboratory of Learning and Memory, Kunming Institute of Zoology, the Chinese Academy of Sciences, Kunming 650223, China.

^2^University of the Chinese Academy of Sciences, Beijing 100049, China.

^3^School of Life Sciences, University of Science and Technology of China, Hefei 230027, China.

^4^School of Life Sciences, Anhui University, Hefei 230601, China.

^5^Institute of Mental Health Research and Departments of Psychiatry and Cellular & Molecular Medicine, University of Ottawa, 1145 Carling Ave, Ottawa, Ontario, K1Z 7K4, Canada.

^6^Sagol Department of Neurobiology and Department of Psychology, University of Haifa, Haifa, Israel.

^7^The Institute for the Study of Affective Neuroscience, University of Haifa, Haifa, Israel.

^8^CAS Center for Excellence in Brain Science, 320 Yue Yang Road, Shanghai, 200031, China.

^9^Mental Health Institute, the Second Xiangya Hospital of Central South University, Changsha 410011, China.

^*^These authors contributed equally to this work.

^#^Corresponding authors: [lxu@vip.163.com](mailto:lxu@vip.163.com) (L.X.); [qixin_zhou@126.com](mailto:qixin_zhou@126.com) (Q-X. Z); [galrichterlevin@gmail.com](mailto:galrichterlevin@gmail.com) (G. R-L).

**
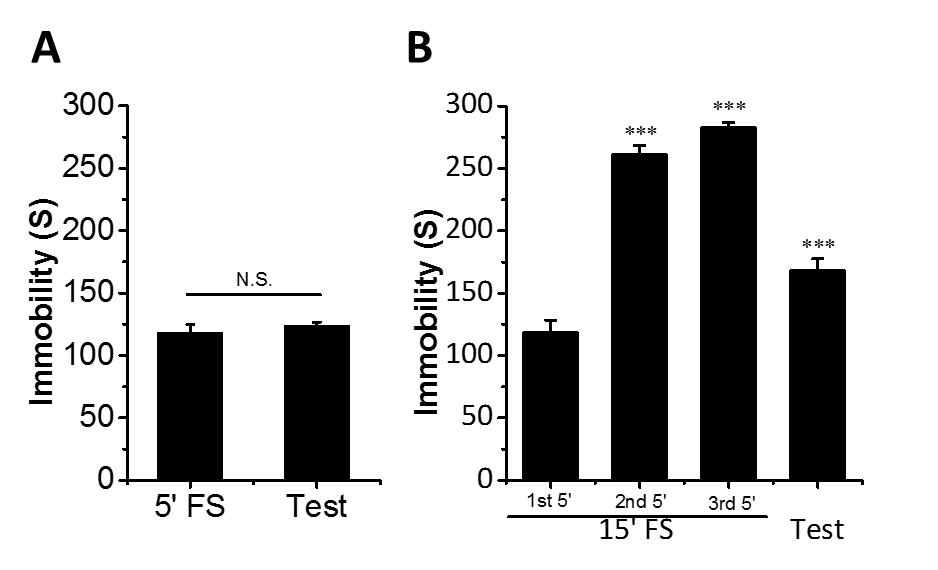
**

**Supplementary figure 1: Forced swimming for 15 min but not 5 min increased test immobility.** A, n = 12, 5’FS = 117.8 ± 6.9 s vs. test = 123.5 ± 3.5 s, *p* = 0.45. B, n = 12, 15’ FS: 1^st^ 5’ (BL) = 118.5 ± 9.9 s, 2^nd^ 5’ = 361.2 ± 7.1 s, 3^rd^ 5’ = 282.7 ± 4.5 s; test = 168.0 ± 9.5 s; all vs. BL, *p* < 0.001. ****p* < 0.001

**
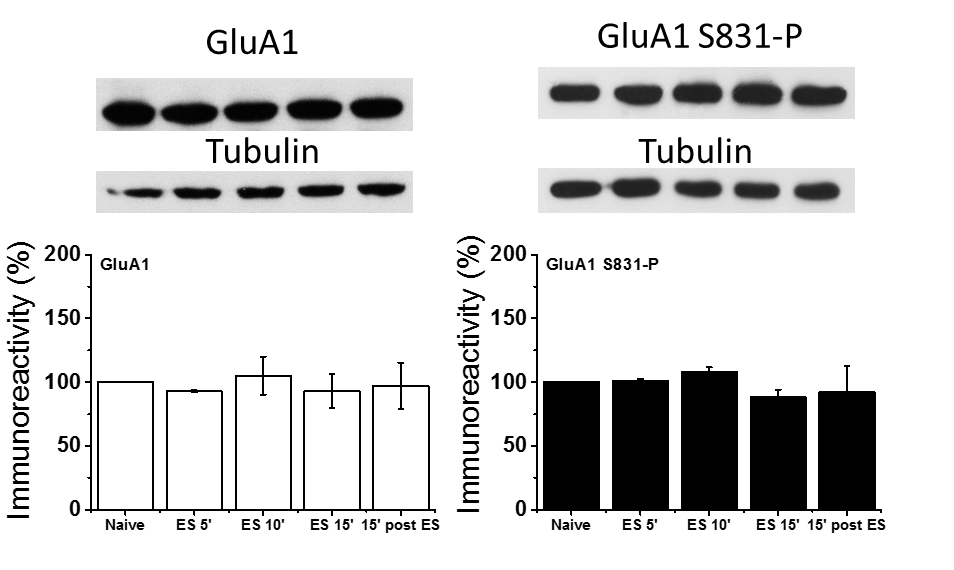
**

**Supplementary figure 2: Escapable swimming (ES) trial had no effect on synaptic GluA1 S831-P in the hippocampus.** Hippocampal synaptoneurosomes were prepared immediately after ES trial for 5-min, 10-min, 15-min (ES 5’; ES 10’; ES 15’) or 15-min post the 15-min ES trial (15’ post ES). Neither GluA1 level nor GluA1 S831-P level was altered by the ES trials. GluA1, 5’ES = 92.9 ± 0.8 %, vs. Naïve, *p* = 0.65; 10’ES = 105.2 ± 14.9 %, vs. Naïve, *p* = 0.74; 15’ES = 93.1 ± 13.2 %, vs. Naïve, *p* = 0.66; 15’post ES = 97.3 ± 18.1 %, vs. Naïve, *p* = 0.88. S831-P, 5’ES = 101.1 ± 1.46 %, vs. Naïve, *p* = 0.91; 10’ES = 108.1 ± 3.6 %, vs. Naïve, *p* = 0.39; 15’ES = 88.6 ± 5.7 %, vs. Naïve, *p* = 0.23; 15’post ES = 92.4 ± 20.1 %, vs. Naïve, *p* = 0.47. For all groups, n = 3.

**
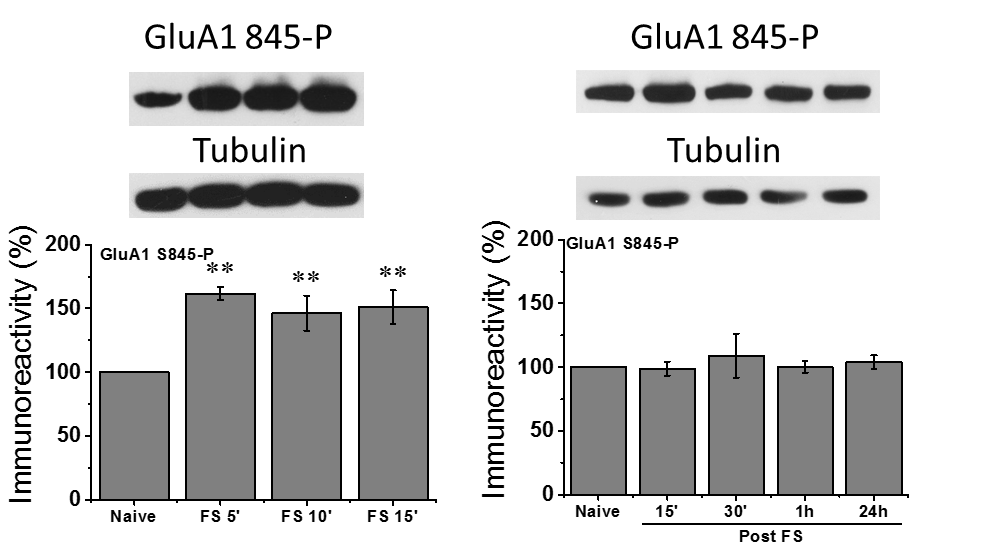
**

**Supplementary figure 3: Synaptic GluA1 S845-P was rapidly increased and then decreased during and after forced swimming (FS).** We prepared the hippocampal synaptic protein during and after the 15-min FS trial. A, S845-P, n = 3; 5’ = 161.7 ± 5.4 %, vs. Naïve, *p* = 0.001 10’ = 146.3 ± 13.7 %, vs. Naïve, *p* = 0.004; 15’ = 151 ± 13.2 %, vs. Naïve, *p* = 0.002. B, post-FS, S845-P, n = 4; 15’ = 98.9 ± 5.4 %, vs. Naïve, *p* = 0.93; n = 5, 30’ = 108 ± 17.3 %, vs. Naïve, *p* = 0.42; 1h = 100.3 ± 4.5 %, vs. Naïve, *p* = 0.98; 24h = 103.8 ± 5.3 %, vs. Naïve, *p* = 0.72. ***p* < 0.01

**Supplementary figure 4: The forced swimming (FS)-increased test immobility was absent in KN62 treated animals compared with vehicle treatment.** 30-min before FS trial, animals were treated with vehicle (VEH) or KN62 via intrahippocampal injection. The immobility during the first 5-min of FS trial and during the test trial was counted. Only VEH group exhibited significant increase of immobility during test trial. VEH group, n = 12, FS trial = 76.7 ± 3.7 s, test trial = 130.5 ± 7.4 s, FS trial vs. test trial, *p* < 0.001. KN62 group, n = 12, FS trial = 46.9 ± 3.8 s, test trial = 46.1 ± 5.1 s, FS trial vs. test trial, *p* = 0.44. *** *p* < 0.001, N.S. not significant.


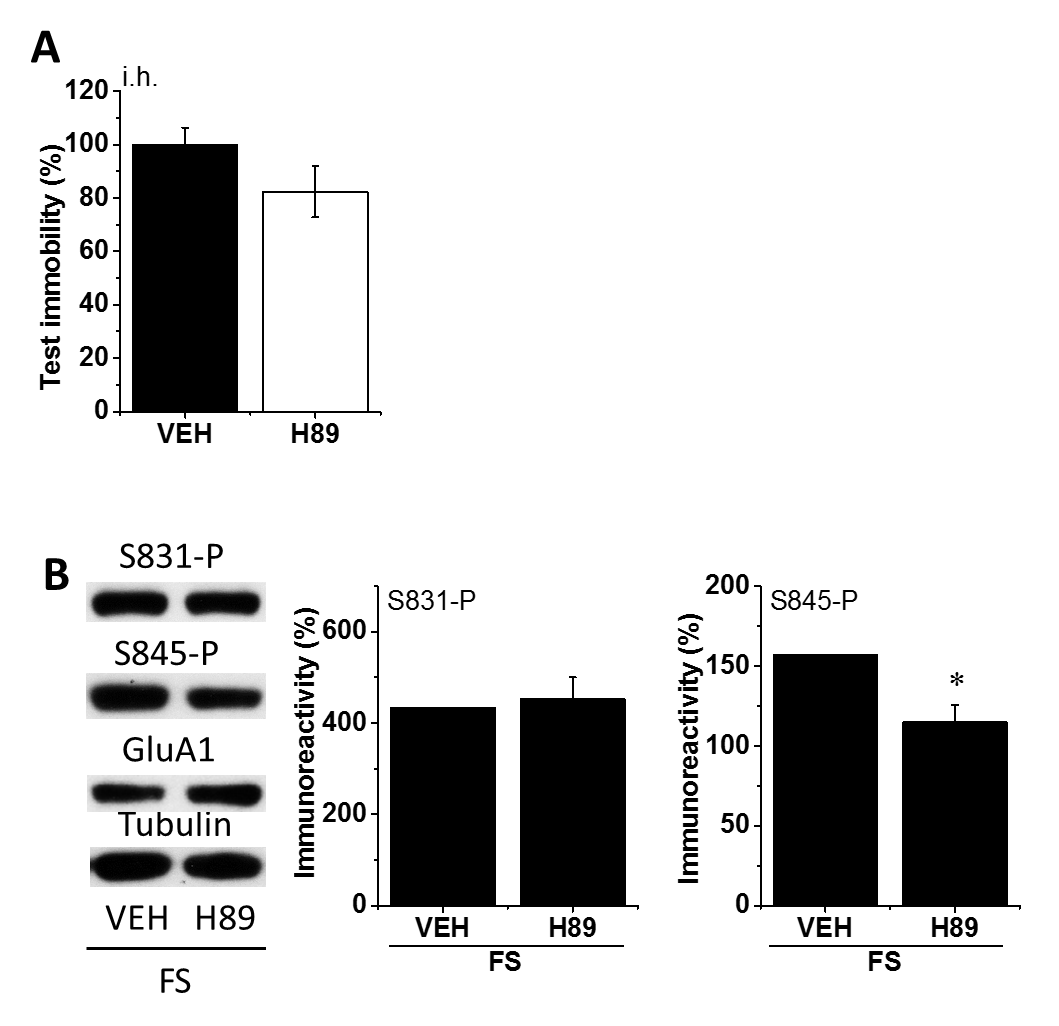


**Supplementary figure 5: Intrahippocampal injection of PKA inhibitor H89 did not affect test immobility and FS-induced S831-P. (**A) Intrahippocampal injection (i.h., 10 μM **,** 1μL/side, 30min before FS trial) of the PKA antagonist H89 had no effect on test immobility, VEH, n = 12, test = 100 ± 6.4 %; H89, n = 11, test = 82.4 ± 9.6 %, VEH vs. H89, *p* = 0.339. **(**B) H89 or VEH was intraperitoneal injected 30 min before FS trial (10 mg/kg). After FS trial, hippocampal synaptoneurosomes were immediately prepared, and then the synaptic GluA1, S831-P, or S845-P were examined. VEH + FS group is set at the level of that in Fig. 3A. S831-P, n = 3; H89 + FS = 452.8 ± 47.24 %, vs. VEH + FS (433.4), *p* = 0.51; S845-P, n = 3; H89 + FS = 114.8 ± 11 %, vs. VEH + FS (156.6), *p* = 0.02. **p* < 0.05


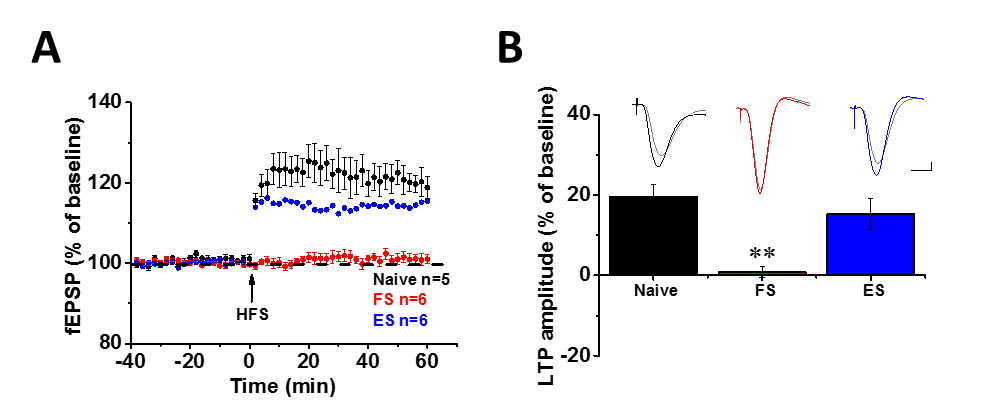


**Supplementary figure 6: High frequency stimulation (HFS) induced CA1 LTP in naïve or ES rats, but not in FS rats that showed increase of test immobility.** After the test trial, rats were anaesthetized for the study of HFS-induced LTP in the hippocampal CA1 area ((HFS, 200 Hz, 10 trains, 20 pulses/train, inter-train interval 2 s). n = 5, Naïve = 119.5 ± 3.2 %; n = 6, FS = 100.9 ± 1.4 %; Naïve vs. FS, p = 0.001; n = 6, ES = 115.3 ± 4.0 %; Naïve vs. ES, p = 0.38; Test immobility: FS = 100 ± 6.0 %; ES = 53.8 ± 5.1 %, FS vs. ES, p < 0.001. ***p* < 0.01
